# Child and Adolescent Virtual Mental Health Care and Duration of Treatment: Retrospective Cohort Study

**DOI:** 10.2196/70650

**Published:** 2025-09-11

**Authors:** Allyson Cruickshank, Pantelis Andreou, Debbie Johnson Emberly, Sandra Meier, Leslie Anne Campbell

**Affiliations:** 1 Department of Community Health and Epidemiology, Faculty of Medicine, Dalhousie University, 5790 University Avenue, Halifax, NS, B3H 1V7, Canada, 1 902-494-3860; 2 Department of Psychology and Neuroscience, Faculty of Science, Dalhousie University, Halifax, NS, Canada; 3 Department of Psychiatry, Faculty of Medicine, Dalhousie University, Halifax, NS, Canada

**Keywords:** child and adolescent mental health, virtual care, episodes of care, mental health services, health services research, telemedicine

## Abstract

**Background:**

Due to public health restrictions, the COVID-19 pandemic required significant changes in the delivery of child and adolescent mental health services. The use of virtual care for balancing access with treatment needs requires a shared decision between clients, caregivers, and clinicians. One aspect for consideration is the length of treatment necessary to achieve desired outcomes and whether it differs by treatment modality. Insights gained from the comparison of treatment duration between modalities may improve our understanding of the effectiveness of virtual care and help to inform clinical decision-making and effective use of resources.

**Objective:**

We sought to improve our understanding of how treatment modality impacts treatment duration for children and adolescents accessing Community Mental Health and Addictions services at IWK Health following the rapid implementation of virtual care in March 2020. In this study, we aimed to compare the duration of treatment within episodes of care by treatment modality and determine whether client characteristics, system factors, or time period influenced any associations between treatment modality and treatment duration.

**Methods:**

Episodes of care were created using administrative data collected by the IWK Mental Health and Addictions program and used as the unit of analysis. A multilevel mixed-effects negative binomial model and time-to-event analysis were used to model the association between treatment modality and treatment duration, both in visits and days, adjusting for client and system characteristics.

**Results:**

Virtual episodes of care had more visits than in-person episodes between April 1, 2020, and March 31, 2021 (incidence rate ratio [IRR] 1.59, 95% CI 1.38-1.83), and April 1, 2021, and March 31, 2022 (IRR 1.22, 95% CI 1.10-1.35), whereas between April 1, 2022, and March 31, 2023, virtual episodes of care were associated with fewer visits (IRR 0.82, 95% CI 0.74-0.91). Comparable results were seen for treatment duration in days (2020‐2021: hazard ratio [HR] 0.64, 95% CI 0.54-0.76; 2021‐2022: HR 0.80, 95% CI 0.70-0.90; and 2022‐2023: HR 1.10, 95% CI 0.97-1.25). These differences by time period relative to the onset of the COVID-19 pandemic and switch to virtual care were consistent after adjusting for client and system characteristics.

**Conclusions:**

To our knowledge, this is the first study to examine the association between virtual or in-person treatment modality and treatment duration. While initially longer than in-person episodes of care, both in numbers of visits and length in days, over time the average length of episodes conducted mainly virtually had attenuated. These findings may be due to growing comfort with the technology or client factors not adequately captured in administrative data. This information can be valuable to clinicians, clients, and their families regarding expected treatment timelines and aid in informing service planning.

## Introduction

The COVID-19 pandemic has had a significant impact on the mental health and well-being of many individuals, with young people bearing a disproportionate weight of this impact [1]. With disruptions to daily routines, social isolation, and increased stress and anxiety, many children and adolescents have experienced new or intensified mental health challenges [2]. The need to address youth mental health concerns became amplified during the pandemic, with calls for supports and resources to help young people navigate these unprecedented times [3,4].

The public health restrictions related to the COVID-19 pandemic requiring physical distancing created the need for mental health service delivery to rapidly shift from primarily in-person to primarily virtual modalities to ensure continued access to services [5]. Moving forward, continuing to provide virtual care options in child and adolescent mental health services may offer several benefits, including improved and more equitable access to mental health services. With access to safe, private spaces and necessary technology, virtual care can allow an individual to access mental health services from their home or school and reduce the logistical barriers of attending an in-person appointment, such as transportation, childcare, and missed work [6,7]. Virtual mental health care may offer privacy to those concerned about the stigma associated with entering a mental health clinic. As such, virtual options can help mitigate barriers preventing individuals from accessing mental health care and thus reach people who may have never attended in-person services [7-9].

However, there needs to be consideration of potential trade-offs between access to and convenience of treatment with individual treatment needs and therapeutic considerations. For some clients, there is a potentially therapeutic benefit in organizing oneself and leaving home to attend an in-person appointment. Clinicians’ comfort with or preferences for treatment modality (ie, mode of treatment delivery, such as virtual and in-person care) may also vary. For example, despite existing evidence of effectiveness of many virtual mental health interventions, some clinicians may hold the belief that virtual care is not as effective as in-person care or requires more sessions to achieve similar treatment goals [10-13]. There is a paucity of research evaluating virtual mental health care, particularly studies with experimental comparisons or assessing effectiveness using mental health outcome measures. Some studies featuring synchronous, clinician-led virtual care have shown promising results regarding the effectiveness of virtual care, although they are limited by small sample sizes and the absence of control groups [14-18]. Furthermore, studies to date have not compared the treatment duration of primarily virtual modalities of care with in-person services. While not a direct measure of effectiveness, treatment duration may reflect meaningful differences between modalities in client engagement or therapeutic progress that affect treatment effectiveness and address clinical perceptions that virtual care “takes longer.” Furthermore, understanding treatment duration can assist in planning for resource allocation to meet the needs of those waiting for care, thereby increasing access to care.

In addition to considering potential trade-offs between treatment modalities for individual clients, understanding patterns of service use for in-person compared with virtual care in terms of the duration of treatment (ie, length of episodes of care in visits and in days) is also important for informing clinical shared decision-making and responsive service planning [19-21]. Anticipated treatment duration offers useful information for clients and families entering treatment, and the communication of any differences between treatment modalities would support shared decision-making. Careful consideration of the expected lengths of treatment or numbers of treatment sessions within an episode of care for a given need is also necessary for understanding implications for adequate staffing to ensure availability and effective utilization of resources. Information about how treatment duration may vary by treatment modality could and should be used to guide needs-based planning and clinical decision-making, supporting a client-centered service delivery model in matching modality (in-person or virtual) to the needs and preferences of the children, youth, and families who come for care [22]. Importantly, understanding treatment duration is a key indicator for the management of wait times for access to mental health care and appropriate resource management to meet the need for care.

Models of care in child and adolescent mental health services increasingly aim to address the dynamic mental health care needs of many children and youth, as reflected in expectations of episodic interactions with the health care system and flexibility in service delivery [19,23,24]. Client-centered services aim to meet children and families where they are in their care journey. This may involve 1 or more interactions with services that are matched to the intensity of their needs at a given time and stage of readiness for treatment, reflected by episodic use of health services. Capturing episodes of care, defined as periods of service use during distinct clinically meaningful periods of need, has been used to examine questions related to health care access, service delivery, and system performance [21,23,25-28]. While not necessarily indicative of the quality or appropriateness of treatment on their own, capturing clinically meaningful health service use patterns in the form of episodes of care can offer valuable insights into treatment frequency and duration. There is no single operational definition of an episode of mental health care to be used when analyzing administrative data.

To our knowledge, the relationship between treatment modality and duration of treatment in child and adolescent mental health ambulatory care has not been studied. This study aims to quantify and compare the treatment duration of episodes of care conducted virtually and in-person, while accounting for the effects of individual-level and system-level factors, and time period relative to the onset of the COVID-19 pandemic.

## Methods

### Study Design and Setting

This study used a retrospective cohort design to examine service use patterns of children and adolescents in receipt of ambulatory mental health services between April 1, 2019, and March 31, 2023, using administrative health data collected by the IWK Mental Health and Addictions (MHA) program. The IWK MHA program provides family-centered mental health and addictions care for children and adolescents up to their 19th birthday in Nova Scotia, Canada. Outpatient (ambulatory) services offered are in 3 Community Mental Health and Addictions clinics and in some schools and other community locations in the Halifax region. Elsewhere provincially, outpatient child and adolescent services are offered by Nova Scotia Health. Ambulatory services are provided using the Choice and Partnership Approach (CAPA). Briefly, CAPA is a client-oriented service model that incorporates the active collaboration of clients, families, and clinicians in care planning and evidence-based treatment, demand and capacity management guided by lean thinking, clinical skill matching to client needs, and goal-based outcome monitoring [29]. “Choice” appointments represent the initial clinical contact, in which clients, families, and clinicians jointly decide upon clinical needs and treatment options [29]. Clients going on to treatment do so in “Partnership,” during which most intervention work occurs with a clinician with skills matched to the client’s clinical needs [29]. Clients may exit and return to care based on their needs.

### Data Sources

Administrative health data sources included Meditech registration and Community Wide Scheduling databases held at IWK Health. Abstracted data included client and service information of children and youth who accessed IWK MHA services between April 1, 2018, and January 31, 2024, including client demographic information, clinic location, outpatient appointment dates, visit type (first “Choice” or treatment “Partnership”), modality (virtual or in-person), acuity at intake, and presenting concerns for approximately 11,000 unique clients.

### Measures

#### Episodes of Care

The unit of analysis, episodes of care, was defined as periods of service use with fewer than 90 days between care contacts for Partnership visits. This was based on an IWK MHA policy requiring a new Choice appointment (ie, new treatment episode) following a 90-day absence from the service. Reid et al [19] analyzed administrative data from 3 childhood mental health agencies in Canada over 7 years, comparing various operational definitions of episodes of care. Reid et al [19] recommended that an episode of care be defined as a minimum of 3 visits with no more than 180 days between contact when conducting analyses of childhood mental health administrative data. However, policies, procedures, treatment model, and service use patterns must be considered when defining an episode of care. As such, a new episode was defined as treatment initiated after greater than 90 days without a care contact, based on the IWK policy to require a new assessment after a 90-day absence from treatment. However, as definitions using fewer than 180 days between care contacts have also been used in the literature, we conducted a sensitivity analysis using a 180-day definition.

#### Treatment Duration

The treatment duration of a care episode, the dependent variable, may be captured in terms of numbers of visits or length of time in treatment. We operationalized the measurement of the treatment duration of a care episode as (1) the number of Partnership visits within the episode, and (2) the length of the episode in days.

#### Treatment Modality

##### Overview

The main independent variable of interest was the treatment modality for the majority of the duration of the episode of care, categorized as (1) in-person mental health care, and (2) virtual mental health care (delivered by telehealth and Zoom for Healthcare). Categorization of the treatment modality was intended to reflect the modality used for a clinically meaningful majority of time within an episode. Thresholds were informed by clinical opinions. These rules were devised to ensure that episodes were characterized based on the modality used for a clinically meaningful majority of time. Thresholds were informed by clinical opinions.

##### In-Person Episodes

In-person episodes of care represent episodes of care in which the majority of treatment visits were delivered in person. Episodes of care with a maximum of 10 visits were limited to 1 or fewer virtual visits. For episodes of care with between 11 and 15 visits, a maximum of 2 virtual visits was permitted. For episodes of 16 visits or longer, a maximum of 3 virtual visits was allowed.

##### Virtual Episodes

Virtual episodes of care represent episodes of care in which the majority of treatment visits were delivered virtually. Episodes of care with a maximum of 10 visits were limited to 1 or fewer in-person visits. For episodes of care with between 11 and 15 visits, a maximum of 2 in-person visits were permitted. For episodes of 16 visits or longer, a maximum of 3 in-person visits was allowed.

##### Hybrid Episodes

Episodes that did not fall into either the in-person or virtual episodes of care categories were considered to be hybrid and were not included in our analyses. This also included episodes of care with exactly 2 visits, 1 of each modality. Despite efforts in attempting to further categorize and separate this category to allow for a better understanding of its content, the episodes within this category varied too widely to allow for meaningful comparisons with the in-person and virtual episodes. Therefore, we excluded hybrid episodes from our analysis.

### Covariates

In our analysis, *study ID*, a randomly assigned number, was used to identify unique individuals, while protecting confidentiality. *Age* was categorized into 3 groups representing clinically meaningful client populations: 0‐11 years, 12‐18 years, and 19+ years. Although the IWK MHA program typically provides services to clients younger than 19 years, it is not uncommon that those older than 19 years receive services. This is most common due to those who are transitioning to adult services being seen for bridging while this transition occurs. Additionally, those who are near the end of a treatment episode may be seen beyond their 19th birthday for completion of care, and some specialty services may continue to see clients due to lack of comparable care in adult services. *Sex* (biological) was classified as male or female, as available in the administrative data. *Presenting concern* was based on the primary issue identified at the initial Partnership visit. Presenting concerns are based upon *Diagnostic and Statistical Manual of Mental Disorders* (*Fifth Edition*) (*DSM-V*) diagnostic codes and are assigned by the Choice clinician and updated by the Partnership clinician as needed. The categories of presenting concern included in the model were substance use disorders, mood disorders (mood and bipolar), anxiety disorders, personality disorders, eating and other feeding disorders, neurodevelopmental disorders (attention-deficit/hyperactivity disorder and autism spectrum disorder), neurocognitive disorders (cognitive and developmental delay), trans health, to be determined, other mental health disorders (disruptive behavior, forensic risk, obsessive-compulsive disorder, Tourette syndrome, trauma, and psychosis), and “not available.” *Urgent triage stream* was assigned to episodes for which the first Partnership visit occurred within the urgent triage stream and was used to distinguish from episodes occurring within the nonurgent triage stream. The triage stream (urgent or nonurgent) differs in terms of initial wait time benchmarks (eg, 7 or 28 days) and is assigned based on clinical triage that reflects an assessment of the urgency of need for care. The distinction between *Core* and *Core and Specific Partnership* was made based on whether the episode consisted solely of Core Partnership visits or included both Core and Specific Partnership visits. In the CAPA model, the majority of treatment occurs within the Core pathway, with Specific Partnership work consisting of specialized assessment or treatment added as necessary to meet client needs and goals of treatment. It was anticipated that those with both Core and Specific Partnership visits would have longer episodes of care or have more frequent visits within an episode of care than those with solely Core Partnership care. *Clinic* referred to the location where the client was seen during the initial Partnership visit within an episode of care, which included the 3 outpatient clinics in the Halifax region (Halifax, Dartmouth, and Sackville) and School Mental Health. The *time period* of a given episode was assigned based on the fiscal year (2019/20, 2020/21, 2021/22, and 2022/23) in which more than 50% of the episode occurred. Acuity at presentation to Community Mental Health and Addictions services is measured using the HEADS-ED (Home, Education & Employment, Activities & Peers, Drugs & Alcohol, Suicidality, Emotions, Behaviors, Thought Disturbance, and Discharge or Current Resources) screening tool during intake at the IWK Central Referral service. The HEADS-ED is a validated mental health screening tool used to obtain a psychosocial history and guide decision-making regarding client disposition. A higher overall HEADS-ED score indicated a need for more intensive or immediate mental health services [30,31].

### Analyses

We used a multilevel mixed-effects negative binomial model to examine the association between treatment modality and treatment duration in numbers of visits. This model was chosen due to the hierarchical structure of the data, with episodes of care nested within individual study IDs and the overdispersed count nature of the outcome. Additionally, because the mean treatment duration in visits differed from the variance, a negative binomial model was selected over a Poisson model. An unadjusted model was used to estimate the relationship between treatment modality and treatment duration, and an adjusted model included fixed effects such as age, sex, presenting concern, urgent stream, Specific Partnership, and clinic. We treated the study ID as a random effect. To identify confounders, we compared the measure of association between treatment modality and treatment duration both before and after adjusting for a potential confounding factor, one at a time. If the difference was greater than 10%, we concluded that confounding was present and adjusted for these variables in our analysis [32]. To identify effect measure modification, we calculated stratum-specific measures of association. If the stratum-specific measures were different from one another, the variable was identified as an effect modifier, and the stratum-specific measures of association were reported [33]. As we expected real temporal differences in treatment duration by modality over the observation period, we treated time period (fiscal years 2019/20, 2020/21, 2021/22, and 2022/23) as an effect modifier and reported stratified results accordingly. We excluded outliers; therefore, we restricted the model to episodes shorter than 25 visits, which corresponds to the 95th percentile of the visit distribution. Episodes exceeding 25 visits may differ in client and clinical characteristics and may be more likely to be classified as hybrid due to their extended duration and increased likelihood of exposure to both modalities.

As a sensitivity analysis to assess the impact of treatment modality on treatment duration in days, a time-to-event analysis was used. We included client and system characteristics with statistically significant associations and retained modalities that met the proportional hazards assumption. The client characteristics included in the time-to-event analyses were sex and presenting concern. We stratified the results by time period.

### Ethical Considerations

The IWK Health Research Ethics Board approved the overarching research project, including this study (“Our Virtual Reality: Rapidly Responding to Changing Mental Health Needs Among Children and Adolescents,” project number 1026770). Informed consent was not required for this secondary analysis of deidentified administrative data. Members of the research team did not have access to any identifying information for visits captured in the administrative databases.

## Results

Table 1 shows the demographic, clinical, service, and health service use characteristics of episodes of care by time period. With respect to clinical characteristics, anxiety disorders were the most common presenting concerns in period 1 (543/1939, 28.00%) and period 4 (514/1692, 30.38%). The mean HEADS-ED score was the lowest in period 1 (mean 5.40, SD 1.82) and the highest in period 3 (mean 5.99, SD 1.70). In period 3, the proportion of episodes identified as urgent was the highest (84/1139, 7.37%) compared with other periods. The mean number of visits per episode was the longest in period 2 (fiscal year 2020/21) (mean 6.69, SD 6.47) relative to the other periods. Descriptive statistics using the 180-day definition can be found in Table S1 in Multimedia Appendix 1. The characteristics of the episodes of care were not meaningfully different between the 2 definitions.

The incidence rate ratios (IRRs) and 95% CIs comparing treatment duration by treatment modality using the unadjusted multilevel mixed-effects negative binomial model are shown in Table 2. Prior to adjustment for client and service factors, virtual episodes of care had 1.14 times more visits than in-person episodes of care (IRR 1.14, 95% CI 1.09-1.19).

The results of the adjusted multilevel mixed-effects negative binomial model are shown in Table 3. We adjusted for age, sex, presenting concern, urgent stream, Specific Partnership, and clinic and stratified by time period. Among all the periods, period 2 had the greatest number of visits per episode for virtual episodes (IRR 1.59, 95% CI 1.38-1.83; *P*<.001) as compared with in-person episodes. In period 4, virtual episodes of care had fewer visits (IRR 0.82, 95% CI 0.74-0.91; *P*<.001) than in-person episodes of care.

**Table 1.** Descriptive statistics of the full cohort of episodes of care by time period.

	Period 1 (FY^a^ 2019/20; n=1939 episodes, 30%)	Period 2 (FY 2020/21; n=1370 episodes, 21%)	Period 3 (FY 2021/22; n=1435 episodes, 22%)	Period 4 (FY 2022/23; n=1692 episodes, 26%)
Client demographic characteristics				
Age at first visit in episode (years), mean (SD)	13.64 (3.34)	13.64 (3.41)	13.84 (3.12)	13.84 (3.05)
Age category (years), n (%)				
0‐11	502 (25.89)	342 (24.96)	297 (20.70)	359 (21.22)
12‐18	1418 (73.13)	1015 (74.09)	1128 (78.61)	1319 (77.96)
>18	19 (0.98)	13 (0.95)	10 (0.70)	14 (0.83)
Sex, n (%)				
Female	1082 (55.80)	789 (57.59)	883 (61.53)	1042 (61.58)
Male	857 (44.20)	581 (42.41)	552 (38.47)	650 (38.42)
Client clinical characteristics				
Presenting concern, n (%)				
Anxiety disorders	543 (28.00)	261 (19.05)	411 (28.64)	514 (30.38)
Mood disorders	390 (20.11)	146 (10.66)	177 (12.33)	196 (11.58)
Neurodevelopmental disorders	149 (7.68)	55 (4.01)	78 (5.44)	121 (7.33)
Substance use and related disorders	112 (5.78)	61 (4.45)	47 (3.28)	61 (3.61)
Trans health	77 (3.97)	59 (4.31)	48 (3.34)	49 (2.90)
Personality disorders	9 (0.46)	6 (0.44)	10 (0.70)	19 (1.12)
Eating and other feeding disorders	18 (0.93)	24 (1.75)	33 (2.30)	24 (1.42)
Neurocognitive disorders	<5 (<1)	<5 (<1)	<5 (<1)	5 (<1)
Other mental health disorders	305 (15.73)	168 (12.26)	211 (14.70)	220 (13.00)
To be determined	9 (0.46)	449 (32.77)	416 (28.99)	477 (28)
Not available	323 (16.66)	140 (10.22)	1 (<1)	6 (0.35)
HEADS-ED^b^ score, n	797	532	582	673
Mean (SD)	5.40 (1.82)	5.78 (1.68)	5.99 (1.70)	5.77 (1.72)
Service characteristics				
Urgent stream, n (%)				
Urgent	60 (3.09)	37 (3.36)	84 (7.37)	60 (4.50)
Nonurgent	1879 (96.91)	1065 (96.64)	1055 (92.63)	1272 (95.50)
Specific Partnership, n (%)				
100% Core	1767 (91.13)	1165 (85.04)	1225 (85.37)	1422 (84.04)
Core and Specific	172 (8.87)	205 (14.96)	210 (14.63)	270 (15.96)
Clinic, n (%)				
Dartmouth	471 (24.29)	321 (23.43)	375 (26.13)	380 (22.46)
Halifax	542 (27.95)	372 (27.15)	320 (22.30)	424 (25.06)
Sackville	459 (23.67)	275 (20.07)	284 (19.79)	371 (21.93)
School	467 (24.08)	402 (29.34)	456 (31.78)	517 (30.56)
Health service use characteristics				
Number of visits per episode, mean (SD)	6.28 (5.47)	6.69 (6.47)	6.23 (6.48)	6.48 (5.73)
Episode length in days, mean (SD)	122.48 (120.74)	112.97 (121.79)	113.39 (132.52)	118.39 (113.80)
Episode modality, n (%)				
In-person	1931 (99.59)	240 (17.52)	552 (38.47)	1279 (75.59)
Virtual	8 (0.41)	1130 (82.48)	883 (61.53)	413 (24.41)

a FY: fiscal year.

b HEADS-ED: Home, Education & Employment, Activities & Peers, Drugs & Alcohol, Suicidality, Emotions, Behaviors, Thought Disturbance, and Discharge or Current Resources.

**Table 2.** Unadjusted multilevel mixed-effects negative binomial model for treatment duration in visits.

	IRR^a^	95% CI	*P* value
Treatment modality (reference: in-person)			
Virtual	1.14	1.09-1.19	<.001
Constant	5.04	4.91-5.17	<.001
Variance between individuals	0.13	0.11-0.17	—^b^

a IRR: incidence rate ratio.

b Not applicable.

**Table 3.** Adjusted multilevel mixed-effects negative binomial model for treatment duration in visits.

Characteristics	Period 1 (FY^a^ 2019/20)		Period 2 (FY 2020/21)		Period 3 (FY 2021/22)		Period 4 (FY 2022/23)	
	IRR^b^ (95% CI)	*P* value	IRR (95% CI)	*P* value	IRR (95% CI)	*P* value	IRR (95% CI)	*P* value
Treatment modality (reference: in-person)								
Virtual	0.33 (0.16-0.68)	.003	1.59 (1.38-1.83)	<.001	1.22 (1.10-1.35)	<.001	0.82 (0.74-0.91)	<.001
Age (years) (reference: 0‐11)								
12‐18	1.08 (0.97-1.19)	.16	1.14 (1.01-1.28)	.03	1.02 (0.90-1.16)	.72	0.98 (0.88-1.09)	.71
>18	0.47 (0.30-0.73)	.001	0.38 (0.17-0.89)	.03	1.10 (0.37-3.33)	.86	N/A^c^	N/A
Sex (reference: male)								
Females	1.03 (0.95-1.11)	.50	1.10 (0.99-1.21)	.07	1.07 (0.97-1.19)	.19	1.11 (1.01-1.21)	.03
Presenting concern (reference: substance use and related disorders)								
Anxiety disorders	1.35 (1.13-1.62)	.001	1.38 (1.07-1.79)	.02	1.66 (1.23-2.23)	.001	1.62 (1.27-2.07)	<.001
Mood disorders	1.37 (1.14-1.65)	.001	1.41 (1.07-1.87)	.02	1.79 (1.32-2.44)	<.001	1.51 (1.17-1.95)	.001
Neurodevelopmental disorders	1.11 (0.89-1.38)	.36	1.27 (0.90-1.79)	.18	1.57 (1.11-2.23)	.01	1.49 (1.13-1.96)	.004
Trans health	0.86 (0.65-1.15)	.31	0.91 (0.62-1.33)	.62	1.31 (0.84-2.03)	.23	0.87 (0.57-1.33)	.52
Personality disorders	1.37 (0.78-2.40)	.27	1.25 (0.61-2.59)	.54	1.30 (0.71-2.40)	.39	1.38 (0.86-2.20)	.18
Eating and other feeding disorders	1.92 (1.29-2.86)	.001	1.64 (1.09-2.48)	.02	1.90 (1.23-2.94)	.004	1.75 (1.12-2.75)	.02
Neurocognitive disorders	0.61 (0.25-1.49)	.28	2.55 (0.60-9.92)	.21	1.31 (0.48-3.59)	.60	1.38 (0.67-2.86)	.90
Other mental health disorders	1.19 (0.97-1.45)	.09	1.21 (0.92-1.59)	.18	1.57 (1.16-2.13)	.004	1.40 (1.09-1.80)	.009
To be determined	0.96 (0.54-1.71)	.89	1.12 (0.87-1.45)	.39	1.99 (1.46-2.71)	<.001	1.36 (1.06-1.73)	.01
Not available	1.12 (0.93-1.36)	.24	1.26 (0.95-1.66)	.10	0.92 (0.16-5.34)	.93	1.60 (0.73-3.51)	.24
Urgent stream (reference: urgent)								
Nonurgent	0.89 (0.72-1.10)	.27	1.26 (0.96-1.65)	.10	0.79 (0.65-0.95)	.01	0.79 (0.65-0.96)	.02
Specific partnership (reference: core)								
Core and specific	1.49 (1.27-1.74)	<.001	1.57 (1.31-1.88)	<.001	1.77 (1.49-2.09)	<.001	1.74 (1.53-1.98)	<.001
Clinic (reference: Dartmouth)								
Halifax	1.06 (0.96-1.18)	.26	0.90 (0.78-1.05)	.20	1.02 (0.89-1.18)	.75	0.99 (0.88-1.12)	.91
Sackville	1.28 (1.14-1.43)	<.001	1.17 (1.00-1.37)	.05	1.13 (0.98-1.31)	.09	1.13 (1.00-1.27)	.05
School	1.05 (0.93-1.18)	.42	0.86 (0.74-0.99)	.04	0.81 (0.70-0.94)	.005	1.03 (0.91-1.17)	.66
Constant	4.30 (3.21-5.74)	<.001	2.07 (1.41-3.04)	<.001	3.02 (2.14-4.26)	<.001	4.43 (3.19-6.16)	<.001
Variance between individuals	0.15 (0.09, 0.25)	—^d^	0.26 (0.14-0.49)	—	0.26 (0.16-0.41)	—	0.20 (0.08-0.46)	—

a FY: fiscal year.

b IRR: incidence rate ratio.

c Not available.

d Not applicable

The results of the Cox Proportional Hazards model were in keeping with those of the negative binomial model. The results can be found in Table S2 in Multimedia Appendix 1. Briefly, the Cox Proportional Hazards model indicated a statistically significant lower hazard ratio (HR 0.64, 95% CI 0.54-0.76; *P*<.001) for the virtual modality than in-person care in period 2, reflecting the shorter length of in-person episodes in days compared with virtual episodes of care. In period 4, the HR was higher (HR 1.10, 95% CI 0.97-1.25; *P*=.14) for the virtual modality but was not statistically significant.

## Discussion

### Principal Findings

In this retrospective cohort study of administrative health data in a large child and adolescent ambulatory mental health service, we studied duration of treatment by episodes of care for virtual and in-person treatment modalities following the rapid implementation of virtual care during the COVID-19 pandemic. When considering episodes of care in terms of the number of visits, the observation that “virtual care takes longer” appeared to be true in the first year following the onset of the pandemic. The duration of treatment was longest during period 2 (fiscal year 2020/21) in terms of numbers of visits per episode of care, attenuating during the following 2 fiscal year periods of observation. Episodes of care delivered virtually in 2020/21 involved nearly 60% more visits than those delivered in person. However, by 2022/23, virtual care episodes involved 18% fewer visits than in-person episodes. When looking at the treatment duration in numbers of days per episode of care, these patterns were not significantly different.

While we accounted for key client and system factors in our analysis, there could be other explanations for our findings that are not available in administrative data. The period between April 1, 2020, and March 31, 2021 (period 2), represents the beginning of the COVID-19 pandemic–related public health restrictions and the rapid implementation of virtual mental health care. Operating mental health services through a virtual medium was relatively new during this time [5,8,34], including in the study clinics in which virtual care was very limited in use prior to the pandemic [7]. Therefore, this required adjustments and adapting to this new service delivery method for both clinicians and clients. Clinicians had to learn how to use virtual care and work through this new virtual medium; it took time to develop these skills [34]. Furthermore, clinicians had to review safety considerations pertaining to using virtual care with the client, which took up time within an appointment, and thus, it took longer to get to the work of identifying treatment goals and providing treatment [34]. Moreover, clients had to adjust to this new way of receiving care [35]. Furthermore, within the first year of the pandemic, the proportion of clients referred to the urgent triage stream increased, as did the average HEADS-ED score [36], which may reflect greater treatment needs and, thus, may be why we observed longer duration of treatment during this time. Perez et al [37] found that during the COVID-19 pandemic, youth attended more virtual care appointments, likely due to increased accessibility and increased mental health needs. However, these virtual sessions were often shorter than in-person sessions, possibly due to the more frequent visits [37]. This may help explain why we observed longer episodes of care during this time.

While our measures of clinical presentation and acuity did not affect our findings, there are likely important client factors that affect service need and attendance but are not adequately captured in administrative data. For example, at the onset of the pandemic, with public health restrictions in place, the availability of in-person treatment was limited; therefore, in-person appointments were held only in cases in which there were safety concerns. As such, those receiving in-person care during this time may have been systematically different from those receiving virtual care [34,38]. Together, these considerations could contribute to virtual episodes of care initially being longer or consisting of more visits while adjusting to this new way of care.

When restrictions were lifted and choice was available, clients, caregivers, and clinicians may have continued using the virtual modality in part due to concerns regarding the risk of contracting COVID-19 disease [7]. However, in period 4, our most recent period, from April 1, 2022, to March 31, 2023, we observed fewer virtual episodes of care (n=413) during this time compared with the start of the COVID-19 pandemic (n=1130). Again, those choosing virtual care during this time may be systematically different in terms of their presenting concerns and treatment needs, which may for the majority be less acute and less complex than those choosing in-person care, and thus, virtual episodes of care during this time may be shorter [38]. Furthermore, due to virtual care having been established for at least 2 years by this time, the efficiency and proficiency in using technology, as well as more efficient and accessible processes and policies, had likely improved for both clients and clinicians over time [34].

### Strengths and Limitations

Administrative data are an important resource for both research and routine quality improvement activities, and this study benefits from the use of a rich administrative dataset that captures detailed individual-level clinical and service use information from across a child and youth ambulatory mental health service. These data provide greater detail on care episodes and patients than the more commonly studied administrative health databases (eg, physician billing and hospital discharge abstract databases). However, the use of administrative data within this study presents some limitations regarding the information that is available. For example, the complexity of a client’s case, their goals of treatment, or their reasons for seeking mental health care (which may differ from the preset categories representing clinician-identified clinical presentations) are not captured but may also impact treatment duration. These factors likely impact treatment duration regardless of modality; however, they could interact with modality in several ways. For instance, virtual care may be preferred or avoided, based on client needs or preferences. Other factors, such as the social determinants of health, including socioeconomic status, health literacy, and family status, which would impact engagement in care and barriers to care, would also be useful to help predict treatment trajectories [39,40]. These factors may also vary across treatment modalities, as certain modalities may present or mitigate barriers for certain clients.

Additionally, the number of clinicians involved in an episode of care could also impact treatment duration as the client may have multiple encounters with multiple providers within an episode and contribute to increased treatment duration. While we accounted for the addition of Specific Partnership work, which would likely involve additional clinicians in a client’s care, we did not explicitly capture the number of clinicians involved in treatment. The use of in-person or virtual care may also vary across providers, based on preferences or clinical requirements. Finally, we do not have a way to confirm that the end of a given episode of care represents the clinical closure in which the treatment goals were met, mutual agreement to end care with limited benefit, or whether clients were lost to follow-up. Future studies would benefit from the distinction of clinical or administrative closures of care episodes as well as the inclusion of routine outcome measures to support conclusions regarding the relative effectiveness of virtual care as reflected by treatment length.

### Conclusions

With the advancement of virtual care as a treatment modality following the onset of the COVID-19 pandemic and the evolving client treatment needs, evidenced by changes in patterns of mental health service use and acuity, this area of research has become increasingly important, especially with the risk of virtual care being abandoned as we transition back to prepandemic (or pre public health restrictions) operations and processes, thus reducing equitable access to mental health services.

This research demonstrates that the duration of treatment for virtual care is comparable with in-person care. While virtual episodes of care were longer immediately following the onset of the pandemic, these differences were small, and by the most recent period of observation, virtual episodes of care were shorter. These findings challenge the perception that virtual care routinely takes longer than in-person treatment. Consequently, as virtual and in-person episodes of care showed broadly similar treatment durations, it supports the continuation of virtual mental health services as a treatment option for children and adolescents, ensuring that those who benefit from virtual care can maintain necessary access to services without undue impact on service capacity.

Further research is necessary to better understand how in-person versus virtual treatment modalities contribute to the effectiveness of care in terms of client mental health outcomes and goals of care. Future studies would also benefit from more comprehensive information on individual client characteristics, such as sociodemographic information and measures of complexity, to help explain any observed differences in treatment duration. Furthermore, information on predictors of outcomes may help explain variations in patterns of service use. Additionally, while our study provided information regarding how treatment modality impacts the duration of episodes of care, the efficacy of virtual care requires further examination. Measures of efficacy in the form of routine outcome measures, alongside information regarding expected treatment timelines, would be important in program-level decisions surrounding the continuation of virtual care and, at the clinical level, supporting shared decision-making.

## Supplementary material

10.2196/70650 Multimedia Appendix 1 Supplementary tables including the descriptive statistics for the 180-day definition of treatment episodes and adjusted Cox proportional hazards model for treatment duration in days by time period.

## Abbreviations

CAPA: Choice and Partnership Approach
HEADS-ED: Home, Education & Employment, Activities & Peers, Drugs & Alcohol, Suicidality, Emotions, Behaviors, Thought Disturbance, and Discharge or Current Resources
HR: hazard ratio
IRR: incidence rate ratio
MHA: Mental Health and Addictions
