# Supplementary material for: Child and Adolescent Virtual Mental Health Care and Duration of Treatment: Retrospective Cohort Study
Source: JMIR Ment Health. 2025 Sep 11;12:e70650. doi: 10.2196/70650 (PMC12425424; doi:10.2196/70650)
Supplement: Multimedia Appendix 1 [file mental-v12-e70650-s001.docx]

Table S1. Descriptive statistics, 180-day definition.

|  | | **Period 1**  **(FY^a^ 2019/20)**  **n=1,351 episodes, (29%)** | **Period 2**  **(FY 2020/21)**  **n=1,050**  **episodes, (22%)** | **Period 3 (FY 2021/22)**  **n=1,102**  **episodes, (24%)** | **Period 4**  **(FY 2022/23)**  **n=1,179 episodes, (25%)** |
| --- | --- | --- | --- | --- | --- |
| **Client Demographic Characteristics** | | | | | |
| Age at first visit in episode, mean (SD) | | 13.43 (3.35) | 13.50 (3.51) | 13.75 (3.14) | 13.77 (3.06) |
| **Age category, n (%)** | |  |  |  |  |
|  | 0-11 | 373 (27.61%) | 276 (26.29%) | 242 (21.96%) | 257 (21.80%) |
|  | 12-18 | 967 (71.58%) | 769 (73.24%) | 855 (77.59%) | 919 (77.95%) |
|  | Over 18 | 11 (0.81%) | 5 (0.48%) | 5 (0.45%) | <5 (<1%) |
| **Sex, n (%)** | |  |  |  |  |
|  | Females | 740 (54.77%) | 612 (58.29%) | 677 (61.43%) | 733 (62.17%) |
|  | Males | 611 (45.23%) | 438 (41.71%) | 425 (38.57%) | 446 (37.83%) |
| **Client Clinical Characteristics** | | | | | |
| **Presenting concern, n (%)** | |  |  |  |  |
|  | Anxiety disorders | 382 (28.28%) | 194 (18.48%) | 332 (30.13%) | 376 (31.89%) |
|  | Mood disorders | 269 (19.91%) | 99 (9.43%) | 147 (13.34%) | 135 (11.45%) |
|  | Neurodevelopmental disorders | 103 (7.62%) | 38 (3.62%) | 60 (5.44%) | 84 (7.12%) |
|  | Substance use and related disorders | 79 (5.85%) | 49 (4.67%) | 26 (2.36%) | 38 (3.22%) |
|  | Trans health | 43 (3.18%) | 32 (3.05%) | 26 (2.36%) | 27 (2.22%) |
|  | Personality disorders | 5 (0.37%) | <5 (<1%) | 7 (0.64) | 11 (0.93%) |
|  | Eating and other feeding disorders | 17 (1.26%) | 22 (2.10%) | 27 (2.45%) | 21 (1.78%) |
|  | Neurocognitive disorders | <5 (<1%) | <5 (<1%) | <5 (<1%) | <5 (<1%) |
|  | Other mental health disorders | 211 (15.62%) | 126 (12.00%) | 173 (15.70%) | 151 (12.81%) |
|  | To be Determined | <5 (<1%) | 368 (35.05%) | 301 (27.31%) | 328 (27.82%) |
|  | Not available | 237 (17.54%) | 117 (11.14%) | <5 (<1%) | <5 (<1%) |
| HEADS-ED score, mean (SD) | | 5.39 (1.81)  (n=794) | 5.77 (1.73)  (n=586) | 5.97 (1.66)  (n=604) | 5.68 (1.68)  (n=703) |
| **Service Characteristics** | | | | | |
| **Urgent Stream, n (%)** | |  |  |  |  |
|  | Urgent | 52 (3.85%) | 36 (3.61%) | 65 (6.20%) | 55 (4.85%) |
|  | Non-urgent | 1,299 (96.15%) | 960 (96.39%) | 984 (93.80%) | 1,078 (95.15%) |
| **Specific Partnership, n (%)** | |  |  |  |  |
|  | 100% Core | 1,246 (92.23%) | 899 (85.62%) | 949 (86.12%) | 1,009 (85.58%) |
|  | Core and Specific | 105 (7.77%) | 151 (14.38%) | 153 (13.88%) | 170 (14.42%) |
| **Clinic** | |  |  |  |  |
|  | Dartmouth | 313 (23.17%) | 256 (24.38%) | 297 (26.95%) | 271 (22.99%) |
|  | Halifax | 382 (28.28%) | 272 (25.90%) | 237 (21.51%) | 272 (23.07%) |
|  | Sackville | 344 (25.46%) | 221 (21.05%) | 220 (19.96%) | 287 (24.34%) |
|  | School | 312 (23.09%) | 301 (28.67%) | 348 (31.58%) | 349 (29.60%) |
| **Health Service Use Characteristics** | | | | | |
| Number of visits per episode, mean (SD) | | 6.34 (5.30) | 7.67 (7.23) | 6.77 (6.59) | 6.34 (5.43) |
| Episode length in days, mean (SD) | | 132.77 (128.13) | 143.46 (151.59) | 136.86 (147.42) | 123.44 (118.38) |
| **Episode modality, n (%)** | |  |  |  |  |
|  | In-person | 1,344 (99.48%) | 186 (17.71%) | 417 (37.84%) | 891 (75.57%) |
|  | Virtual | 7 (0.52%) | 864 (82.29%) | 685 (62.16%) | 288 (24.43%) |

^a^FY: fiscal year.

Table S2. Adjusted Cox Proportional Hazards model for treatment duration in days by time period.

|  | | **Period 1**  **(FY^a^ 2019/20)** | | **Period 2**  **(FY 2020/21)** | | **Period 3**  **(FY 2021/22)** | | **Period 4**  **(FY 2022/23)** | |
| --- | --- | --- | --- | --- | --- | --- | --- | --- | --- |
|  | | HR (95% CI) | *P* | HR (95% CI) | *P* | HR (95% CI) | *P* | HR (95% CI) | *P* |
| **Modality** | |  |  |  |  |  |  |  |  |
|  | In-person (reference) |  |  |  |  |  |  |  |  |
|  | Virtual | 0.44 (0.13, 1.44) | .174 | 0.64 (0.54, 0.76) | <.001 | 0.80 (0.70, 0.90) | <.001 | 1.10 (0.97, 1.25) | .141 |
| **Sex** | |  |  |  |  |  |  |  |  |
|  | Males (reference) |  |  |  |  |  |  |  |  |
|  | Females | 1.00 (0.90, 1.12) | .939 | 0.96 (0.84, 1.08) | .485 | 0.89 (0.78, 1.00) | .058 | 0.88 (0.78, 0.98) | .025 |
| **Presenting Concern** | |  |  |  |  |  |  |  |  |
|  | Substance use and related disorders (reference) |  |  |  |  |  |  |  |  |
|  | Anxiety disorders | 0.64 (0.50, 0.80) | <0.001 | 0.80 (0.56, 1.13) | .204 | 0.43 (0.30, 0.63) | <.001 | 0.67 (0.49, 0.92) | .013 |
|  | Mood disorders | 0.69 (0.54, 0.87) | .002 | 0.89 (0.61, 1.29) | .540 | 0.35 (0.24, 0.53) | <.001 | 0.73 (0.52, 1.02) | .061 |
|  | Neurodevelopmental disorders | 0.83 (0.63, 1.10) | .198 | 0.75 (0.48, 1.17) | .202 | 0.52 (0.33, 0.79) | .003 | 0.76 (0.53, 1.07) | .114 |
|  | Trans health | 0.77 (0.51, 1.16) | .208 | 0.92 (0.57, 1.47) | .716 | 0.51 (0.30, 0.88) | .015 | 1.01 (0.61, 1.67) | .965 |
|  | Personality disorders | 0.86 (0.42, 1.79) | .695 | 1.62 (0.68, 3.86) | .275 | 0.35 (0.12, 1.00) | .051 | 0.83 (0.48, 1.44) | .506 |
|  | Eating and other feeding disorders | 0.86 (0.50, 1.47) | .57 | 0.65 (0.38, 1.13) | .128 | 0.54 (0.32, 0.91) | .022 | 0.90 (0.53, 1.52) | .686 |
|  | Neurocognitive disorders | 2.66 (0.65, 10.87) | .173 | 1.01 (0.14, 7.46) | .988 | 0.47 (0.11, 1.97) | .299 | 0.68 (0.21, 2.22) | .529 |
|  | Other mental health disorders | 0.77 (0.60, 0.98) | .037 | 0.99 (0.70, 1.41) | .956 | 0.46 (0.31, 0.68) | <.001 | 0.86 (0.62, 1.20) | .373 |
|  | To be Determined | 0.65 (0.30, 1.41) | .275 | 1.04 (0.74, 1.46) | .824 | 0.35 (0.24, 0.52) | <.001 | 0.76 (0.56, 1.04) | .089 |
|  | Not available | 0.91 (0.71, 1.17) | .456 | 0.86 (0.59, 1.25) | .418 | 2.38 (0.32, 17.99) | .4 | 1.70 (0.67, 4.30) | .263 |

^a^FY: fiscal year.
